# High-throughput bioprinting to produce micropatterned neuroepithelial tissues and model TSC2-deficient brain malformations

**DOI:** 10.1016/j.crmeth.2025.101177

**Published:** 2025-09-17

**Authors:** Negin Imani Farahani, Kenneth Kin Lam Wong, George Allen, Abhimanyu Minhas, Lisa Lin, Shama Nazir, Lisa M. Julian

**Affiliations:** 1 Department of Molecular Biology and Biochemistry, Simon Fraser University, 8888 University Drive, Burnaby, BC V5A 1S6, Canada; 2 Department of Biological Sciences, Simon Fraser University, Burnaby, BC V5A 1S6, Canada; 3 Centre for Cell Biology Development and Disease, Simon Fraser University, Burnaby, BC V5A 1S6, Canada; 4 Institute for Neuroscience and Neurotechnology, Simon Fraser University, Burnaby, BC V5A 1S6, Canada

**Keywords:** micropatterning, bioprinting, scaffolded neuroepithelial tissues, neuruloids, human pluripotent stem cells, neurodevelopment, neural tube, tuberous sclerosis complex, cortical malformations, cortical folding

## Abstract

*In vitro* human pluripotent stem cell (hPSC)-derived models have been crucial in advancing our understanding of the mechanisms underlying neurodevelopment, though knowledge of the earliest stages of brain formation is lacking. Micropatterning of cell populations as they transition from pluripotency through the process of neurulation can produce self-assembled neuroepithelial tissues (NETs) with precise spatiotemporal control, enhancing the fidelity of hPSC models to the early developing human brain and their use in phenotypic assessments. Here, we introduce an accessible, customizable, and scalable method to produce self-assembled NETs using bioprinting to rapidly deposit reproducibly sized extracellular matrix droplets. Matrix addition to the media provides a scaffold that promotes 3D tissue folding, reflecting neural tube development. We demonstrate that these scaffolded NETs (scNETs) exhibit key architectural and biological features of the human brain during normal and abnormal development—notably, hyperproliferation and structural malformations induced by *TSC2* deficiency—and provide a robust drug screening tool.

## Highlights

• Bioprinting yields hPSC-neuroepithelial tissue (NET) micropatterns in high throughput
• Matrigel promotes 3D folding into neural tube-like scaffolded NETs (scNETs) in 6 days
• Self-assembled scNETs exhibit natural apicobasal polarity around a single lumen
• *TSC2*^−/−^ scNETs show rapamycin-sensitive excess proliferation and cortical folding

## Motivation

Micropatterning technology to spatially guide the self-assembly of human pluripotent stem cells (hPSCs) into neural tissues has advanced our ability to model early stages of human brain development. Current approaches for hPSC micropatterning rely on specialized materials that limit accessibility and throughput. To address these limitations, we developed a simple and scalable bioprinting-based approach to generate micropatterned, scaffolded neuroepithelial tissues with a single lumen in 6 days.

## Graphical abstract

**Figure:**
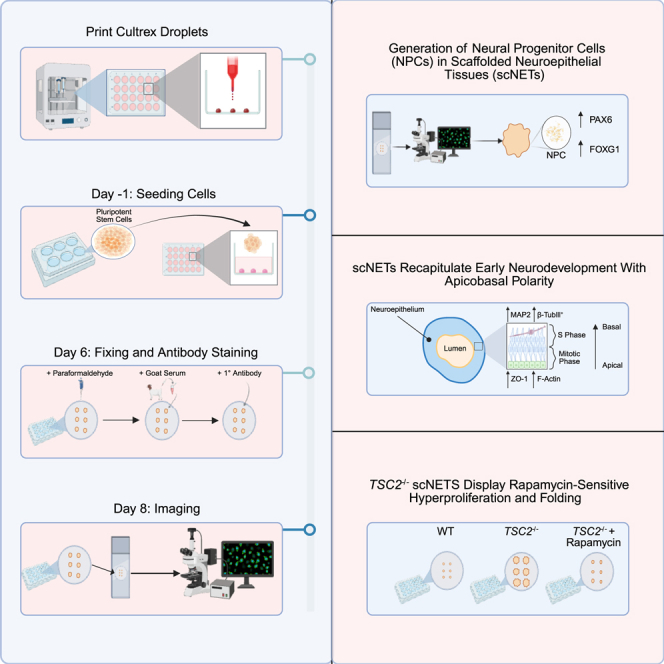

## Teaser

Imani Farahani et al. present a customizable and scalable method to grow neural tube-like tissues from human pluripotent stem cells using a 3D bioprinter. This approach reveals clinical features seen in patients with tuberous sclerosis, offering a high-throughput screening platform to identify disease-causing genes and accelerate drug discovery.

## Introduction

*In vitro* neural cell and organoid models derived from human pluripotent stem cells (hPSCs) are transforming our understanding of the normal and disordered brain. However, the brain’s earliest stage—neurulation—through which the neuroepithelium forms and produces the neural tube, is severely understudied in humans. Disruptions of neurulation can cause neural tube defects (∼2 per 1,000 pregnancies)^1^ and many malformations of cortical development (MCDs), which are a leading cause of refractory epilepsy and altered cognition.^2^^,^^3^^,^^4^ Thus, the advancement of human models is critical to uncover the mechanisms of onset for many neurodevelopmental disorders and to identify pathogenic mutations. Standard two-dimensional (2D) cultures do not suffice, as they lack strict cell density control, resulting in variable cell behaviors,^5^ while 3D organoids are also lacking, as they are heterogeneous, often forming multiple lumens. Micropatterning- or microfluidic-based approaches to produce single-lumen neuroepithelial tissues (NETs) from human induced pluripotent stem cells (hiPSCs) have gained popularity.^6^^,^^7^^,^^8^^,^^9^^,^^10^ These tissues recapitulate structural aspects of the *in vivo* microenvironment by restricting the cell growth surface during neural lineage induction. Current approaches to generate micropatterned tissues, such as photolithography, surface passivation, and contact photo-patterning, however, are often costly, laborious, and low throughput.^11^

Here, we establish a customizable, accessible, and scalable method to produce micropatterned NETs using a bioprinting approach. Inclusion of Matrigel in the culture media provides a scaffold for 3D tissue folding.^8^ We demonstrate that these scaffolded NETs (scNETs) are a physiologically relevant model of both normal and abnormal early neurodevelopment. We produce scNETs from hPSCs carrying inactivating mutations in the *TSC2* gene, which, along with mutations in its functional partner *TSC1*, are the sole cause of the autosomal-dominant multisystem low-grade tumor disorder tuberous sclerosis complex (TSC).^12^ TSC is also highly associated with MCDs, including cortical tubers in 80%–90% of patients, as well as increased gyrification, marked by an abnormal number of cortical folds.^13^^,^^14^ Notably, pathogenic *TSC2* variants account for most (>70%) TSC cases^15^ and are also associated with MCD overgrowth syndromes, including megalencephaly and polymicrogia.^16^^,^^17^^,^^18^^,^^19^^,^^20^^,^^21^ We show that TSC2-deficient scNETs display striking hyperproliferation and tissue malformations that resemble excess cortical folding. Thus, scNETs offer a reproducible and quantifiable model of early MCD formation, and we further demonstrate their potential for high-throughput phenotyping and drug screening applications.

## Results

### Generation of hPSC-derived scNETs by micropatterning on bioprinted matrices

**Figure 1:**
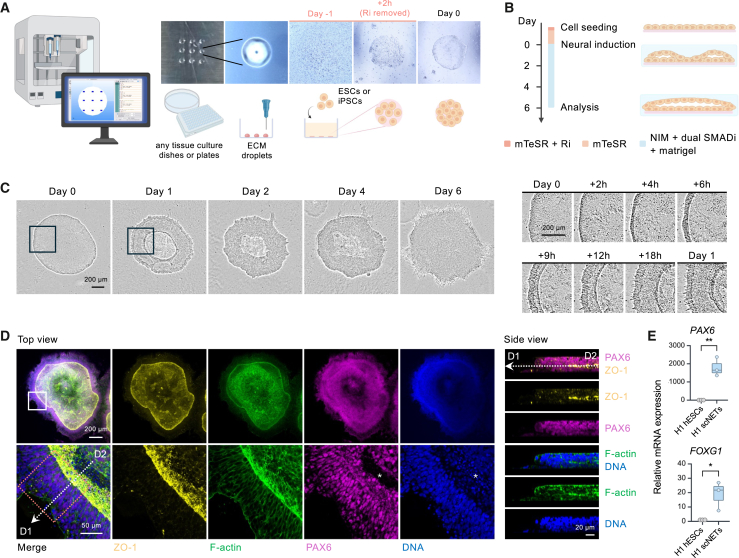
A programmable bioprinting approach to generate hPSC-derived, self-organized, micropatterned scNETs (A) Left to right: 3D bioprinter and G-code for ECM droplet deposition; 9 ECM droplets; single droplet; hPSC seeding; ROCK inhibitor (Ri) removal 2 h post-seeding; hPSC colony formation. (B) Timeline and media for scNET generation, with side views of hPSCs developing into scNETs. (C) Time-lapse imaging of scNET development, with magnified views showing edge thickening (multilayering). See also Video S1. (D) Confocal images of a day 6 scNET (H1) stained for ZO-1 (yellow), F-actin (green), PAX6 (magenta), and DNA (blue). The bottom row shows magnified views of the white boxed inset. See Figure 2A for magnified views of the dashed red inset. The dashed arrow from (D1) to (D2) refers to the *z* section. The asterisk marks a PAX6^−^ but DAPI^+^ region within the lumen, which, given the smaller size of these cells, likely labels fragmented DNA derived from cell debris. See also Video S2. (E) Boxplot shows relative mRNA levels of *PAX6* and *FOXG1* in hESCs (H1) and day 6 scNETs. *n* = 3 biological replicates. ∗∗*p* < 0.005 and ∗*p* < 0.05, unpaired t test. Scale bars: 200 μm, 50 μm, and 20 μm. See also Figures S1 and S2 and Videos S1 and S2.

We set out to develop a scalable physiologically relevant model of early brain development by harnessing hPSCs, neural lineage induction, and bioprinting technologies to generate micropatterned scNETs. We used an extrusion bioprinter to print individual droplets of the extracellular matrix (ECM) basement membrane formulation Cultrex, up to 100 droplets in 3 min, with a defined diameter onto the surface of cell culture vessels (Figure 1A). This enabled spatially restricted growth of hPSCs seeded onto the ECM droplets. We could reliably print droplets as small as 500 μm in diameter (Figure S1A), though 800 μm droplets were used for most experiments, which corresponds to the diameter of the Carnegie stage 13–14 human forebrain neural tube (4–5 gestational weeks).^22^^,^^23^ Highlighting the customizability of our approach, we used G-code, a programming language for 3D bioprinting, to instruct the printer to deposit arrays of droplets with reproducible size and precise spatial arrangements (Figures S1B and S1C). This allows for easy modification of the number, size, shape, and location of droplets to meet the requirements of each experiment. Droplets can be printed on various tissue cultureware, including Petri dishes, multi-well plates, and coverslips (Figure S1D), demonstrating the flexibility, scalability, and high-throughput compatibility of our method. In contrast, manual deposition of ECM droplets, while possible within this size range, is labor intensive and limits spatial precision and throughput (Figure S1F).

Next, we asked if self-organized neural tissues could be produced from hPSCs seeded onto bioprinted matrix droplets by inducing neural differentiation with dual SMAD inhibition.^24^^,^^25^ After seeding, hPSCs attached to and proliferated within the area of printed ECM, forming monolayered, circular colonies (Figure 1A). Once hPSCs reached confluence, neural differentiation was initiated (Figure 1B), marking day 0 of the assay. We included 4% Matrigel in the medium, which, as previously reported,^8^ provides a scaffold for neural folds to project and converge, driving a transition from 2D cultures into 3D single-lumen neural tube-like tissues.

Within 2–4 h of neural induction, multilayering occurred, indicated by the thickening of the micropatterns starting from the edge and progressing inward, and by the end of day 1, a ring-shaped structure had emerged (Figures 1C and S2A; Video S1). The accumulation of filamentous actin (F-actin) between cell layers suggests early spatial organization. This self-organizing process was reproducible across different hPSC lines, including previously described human embryonic stem cells (hESCs: H1) and iPSCs (168), with success rates of 93.8% ± 0.9% (mean ± SEM) in H1 hESC lines and 88.4% ± 6.4% in 168 iPSC lines (Figures S1E and S2B; see method details for the identification of the successful formation of scNETs).^26^^,^^27^ The micropatterned tissues continued to grow and thicken, ultimately forming a circular disk-like structure by day 5–6 (Figure 1D; Video S2). They also developed a central lumen or ventricle-like structure, with its borders defined by enriched ZO-1 (a tight junction scaffolding protein) and F-actin (Figure 1D). Notably, most cells stained positive for PAX6, confirming their transition into neural stem and progenitor cells (NPCs) with a dorsal forebrain identity. This was further corroborated by RT-qPCR analysis, which showed significantly higher expression of *PAX6* and *FOXG1*, another dorsal telencephalon marker, in scNETs compared to undifferentiated hPSCs (Figures 1E and S2C). Gene expression of *NKX2-1* and *EN1*, markers for ventral forebrain and midbrain, respectively, was not detected in hPSCs or scNETs, whereas a significant downregulation of the pluripotency marker *OCT4* was evident (Figure S2C).

Document S1. Figures S1–S4 and Table S1

Video S1. Time-lapse imaging of WT scNET development, related to Figures 1 and S2

### scNETs are a physiologically relevant model for early neurodevelopment

We further investigated the relevance of the scNET model to human neural tube development. As the neural tube forms *in vivo*, neuroepithelial cells become polarized, with the apical domain adjacent to the ventricular lumen and the basal domain extended toward the outer surface of the neural tube.^28^ This apicobasal polarity is crucial for proper interkinetic nuclear migration (IKNM), a process by which the nuclei of NPCs migrate toward the basal surface of the neuroepithelium during G1 to S phases and then back to the apical surface for mitosis.^29^ IKNM plays a vital role in the integrity of cell cycle progression and the fate decisions NPCs make.^30^^,^^31^

**Figure 2:**
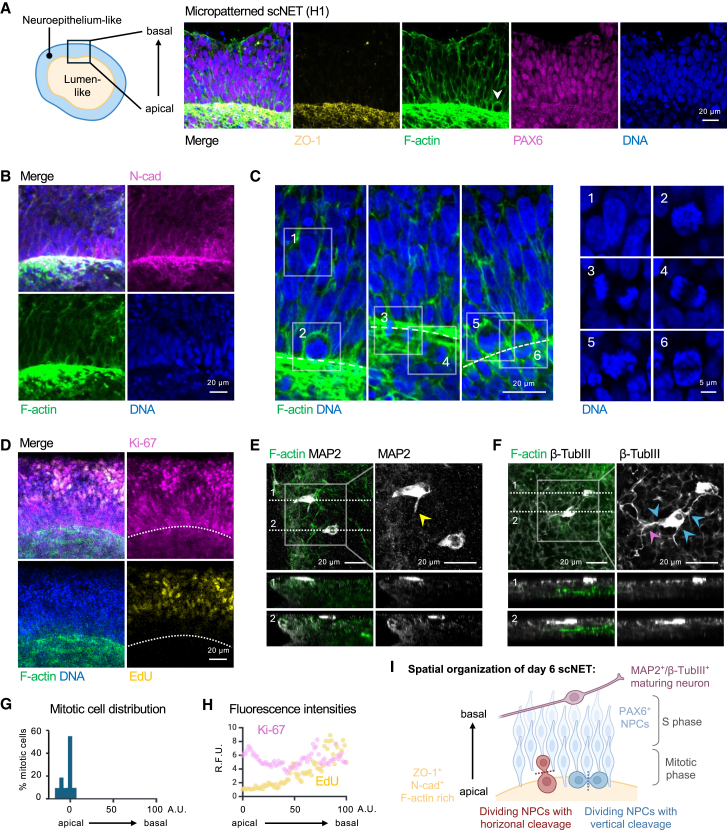
Micropatterned scNETs closely resemble the early neuroepithelium and produce the first neuronal cells within 6 days (A) Schematic of a day 6 scNET (left). Magnified views of the dashed red inset drawn in Figure 1D; ZO-1 (yellow), F-actin (green), PAX6 (magenta), and DNA (blue). Arrowhead marks a PAX6^+^ NPC. (B–D) Confocal images of day 6 scNETs (H1) stained for N-cad (magenta), F-actin (green), and DNA (blue) (B); F-actin (green) and DNA (blue) (C); and Ki-67 (magenta), EdU (yellow), F-actin (green), and DNA (blue) (D). Dashed lines mark the apical surface (C) and (D). (C) Magnified views of the insets (1–6) (right). (E and F) Confocal images show a scNET (H1) stained for MAP2 (gray) and F-actin (green) (E) and a scNET (168) stained for β-TubIII (gray) and F-actin (green) (F). Yellow arrowhead marks a developing neurite. Blue arrowheads mark multiple neurites from the same cell. Purple arrowheads point to neurite branching. Dashed lines refer to the region at which side-view images were taken. (G and H) Distribution graph showing the percentage of mitotic cells across the apicobasal axis of the neuroepithelium layer of scNETs (H1) (G) and scatterplot showing the relative fluorescence units (RFUs) of Ki-67 (purple) and EdU (yellow) across the apicobasal axis (H), using apicobasal distance scaled from 0 to 100 arbitrary units (a.u.), with apical surface marked by 0, basal surface 100, and luminal region negative. (I) Schematic of spatial organization of PAX6^+^ NPCs and MAP2^+^/β-TubIII^+^ maturing neurons in scNETs generated through our bioprinting approach. Scale bars: 20 μm and 5 μm. See also Figure S2.

ZO-1 and F-actin enrichment demarcates the scNET lumen (Figures 1D and 2A). Since ZO-1 marks the apical surface in polarized epithelia,^32^ its localization in the tissues confirmed that PAX6^+^ NPCs had acquired apicobasal polarity, with their apical side facing the center. Enrichment of the apical marker N-cadherin at the lumen side (Figure 2B) further highlights apicobasal polarity. We also observed that although some apical NPCs appeared circular (arrowhead in Figures 2A and 2C2), most were elongated and columnar. This cellular arrangement resembles the pseudostratified neuroepithelium of the developing forebrain, in which NPCs appear multilayered due to the positioning of their nuclei at different heights.^33^^,^^34^

The presence of circular NPCs at the apical surface prompted us to examine the distribution of mitotic cells along the ventricle, as DAPI staining revealed these cells had condensed DNA (Figures 2C2–2C6, S2D, and S2F). Whereas non-dividing cells displayed diffuse DNA (Figure 2C1), the condensed DNA of the dividing cells signified entry into prophase (Figures 2C2 and 2C5). We also identified cells in anaphase, denoted by the separation of sister chromatids toward opposite ends of the cell (Figures 2C3, 2C4, and 2C6), with cleavage planes either perpendicular or parallel to the apical surface (Figure 2I). Notably, mitotic cells were predominantly localized at the apical surface (Figures 2C and 2G), as observed in the native neuroepithelium.^23^^,^^31^

To assess NPC proliferation states, we examined the expression of Ki-67, a nuclear protein that accumulates in proliferating cells and is degraded in quiescence.^35^ Immunostaining showed that Ki-67 was highly expressed in scNETs at both the apical and basal regions (Figures 2D and 2H). 5-ethynyl-2′-deoxyuridine (EdU) labeling, which marks cells replicating their DNA, showed that cells in the DNA synthesis phase of the cell cycle were enriched toward the basal side (Figures 2D and 2H). This spatial distribution of mitotic cells at the apical surface and S-phase cells at the basal side is characteristic of the natural IKNM process.

The orientation of the mitotic cleavage plane is known to infer whether a neural stem cell is undergoing a symmetric self-renewing division or an asymmetric differentiative division.^31^^,^^33^^,^^36^ While most mitotic divisions had a cleavage plane parallel or vertical to the apical surface, suggesting proliferative self-renewing divisions, cells with a horizontal cleavage plane were observed, suggesting a low level of asymmetric divisions that might generate early neurons (Figure 2C). Immunostaining for β-tubulin III (β-TubIII) and MAP2 confirmed the presence of immature and maturing neurons by day 6 in the scNETs (Figures 2E, 2F, and S2G). These cells were localized at the basal side, characteristic of developing neurons *in vivo.**^37^*

Together, these findings demonstrate that PAX6^+^ NPCs within scNETs recapitulate key features of the neuroepithelium, including apicobasal polarity, pseudostratification, IKNM, and early stages of neurogenesis (Figure 2I), making this a highly relevant model to study early corticogenesis.

### scNETs generated from *TSC2*^−/−^ hPSCs display hyperproliferation and cortical folding

**Figure 3:**
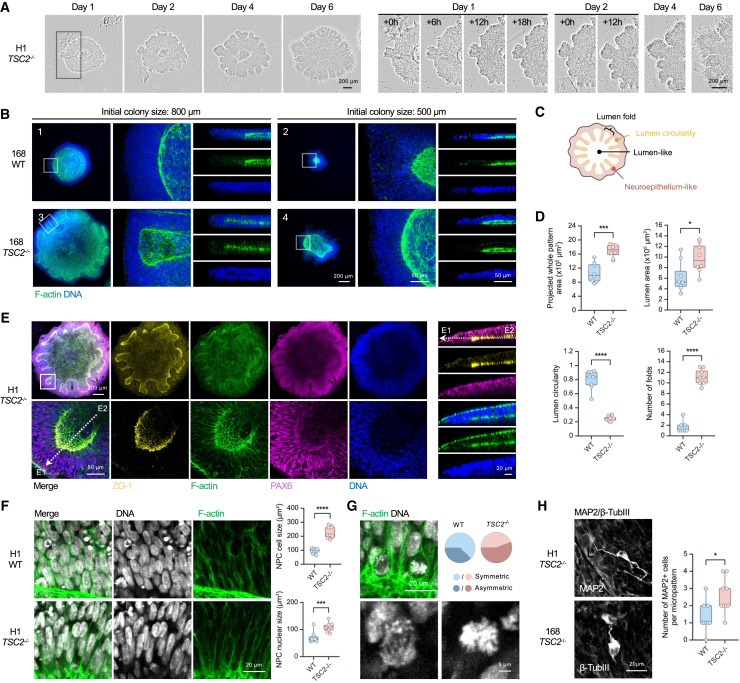
Micropatterned scNETs derived from *TSC2*^−/−^ hPSCs display hyperproliferation and cortical folding phenotypes (A) Time-lapse imaging of *TSC2*^−/−^ scNET development (H1), with magnified views showing the emergence of a cortical folding-like phenotype. See also Video S3. (B) Confocal images of day 6 WT (B1 and B2) and *TSC2*^−/−^ (B3 and B4) scNETs (168) with their indicated initial colony sizes stained for F-actin (green) and DNA (blue). (C) Schematic of *TSC2*^−/−^ scNET with morphological abnormalities. (D) Boxplots showing projected whole pattern area, lumen area, lumen circularity, and number of folds of day 6 WT scNETs (H1) compared with the *TSC2*^−/−^ scNETs. ∗∗∗∗*p* < 0.00005, ∗∗∗*p* < 0.0005, and ∗*p* < 0.05, unpaired t test. (E) Confocal images of a day 6 *TSC2*^−/−^ scNET (H1) stained for ZO-1 (yellow), F-actin (green), PAX6 (magenta), and DNA (blue). See also Video S2. (F) Confocal images of day 6 WT and *TSC2*^−/−^ scNETs (H1) stained for F-actin (green) and DNA (gray). Boxplots showing the quantifications of cell size (outlined by F-actin) and nuclear size. ∗∗∗∗*p* < 0.00005 and ∗∗∗*p* < 0.0005, unpaired t test. (G) Confocal images showing day 6 *TSC2*^−/−^ scNETs (H1) stained for F-actin (green) and DNA (gray). Pie chart shows the percentage of symmetric and asymmetric divisions. (H) Confocal images showing MAP2^+^ and β-TubIII^+^ cells of day 6 *TSC2*^−/−^ scNETs. Boxplot showing the number of MAP2^+^ cells in WT scNETs and in *TSC2*^−/−^ scNETs (H1). ∗*p* < 0.05, unpaired t test. Scale bars: 200 μm, 50 μm, and 20 μm. See also Figure S3 and Videos S2 and S3.

The physiological relevance of scNETs prompted us to ask if disease-associated phenotypes could be identified and quantified in these tissues. We focused on TSC2, which acts in complex with TSC1 to inhibit the nutrient-sensing mammalian target of rapamycin complex 1 (mTORC1) signaling pathway.^38^^,^^39^ Inactivating mutations in *TSC2* result in MCDs due to excess cell proliferation and altered differentiation, largely due to mTORC1 hyperactivation.^4^^,^^17^^,^^39^^,^^40^^,^^41^^,^^42^ Thus, we generated scNETs using *TSC2-*deficient hPSCs (*TSC2*^−/−^) that were previously engineered by CRISPR-Cas9 genome editing.^26^^,^^27^ We first confirmed the knockout mutation in these cells via PCR (Figure S3A). One day after neural induction, the developing *TSC2*^−/−^ scNETs were indistinguishable from the wild type (WT), characterized by the formation of a standard ring-like structure (Figure 3A, versus control in Figure 1C). However, as early as day 2, unlike WT, *TSC2*^−/−^ scNETs began to develop a scalloped border that is suggestive of cortical folding. Excess cortical folds or gyri have been observed in TSC2-driven MCDs, including TSC and polymicrogyria,^14^^,^^17^^,^^43^ though this phenotype has not been modeled in human organoids. These convex folds became more prominent as the *TSC2*^−/−^ scNETs continued to grow (Figure 3A) and were observed in both H1 ESC- and 168 iPSC-derived scNETs (Figures S3B and S3C).

Video S2. 3D fluorescence visualization of day 6 scNETs, related to Figures 1 and 3

Taking advantage of the flexibility of our bioprinting approach to modify ECM droplet size, we asked if *TSC2*^−/−^ phenotypes would be affected by initial colony size. WT scNETs grown on 500 μm ECM droplets were smaller than those grown on 800 μm droplets (Figures 3B1 and 3B2). Both featured a central lumen structure. Interestingly, whereas *TSC2*^−/−^ scNETs grown on 800 μm ECM islands displayed extensive folding, those on 500 μm developed fewer folds, indicated by the lumen circularity (Figures 3B3 and 3B4). Yet, the *TSC2*^−/−^ scNETs grown on 500 μm ECM droplets were still larger than their control counterparts (Figures 3B2 and 3B4). These data suggest the *TSC2*^−/−^ hyperproliferative phenotype occurs regardless of the initial colony size but that the folding phenotype occurs in larger *TSC2*^−/−^ scNETs, possibly because a larger colony size may provide more room for the cells to grow and interact with one another.

PAX6^+^ NPCs were abundant in *TSC2*^−/−^ scNETs, like the WT, and displayed apicobasal polarity with the apical side marked by ZO-1 and differentiated into MAP2^+^/β-TubIII^+^ cells with visible neurites (Figures 3E–3H). We noted a significant increase in cell size in *TSC2*^−/−^ scNETs (Figure 3F), in line with previous reports.^44^^,^^45^ Our analysis further revealed increased asymmetric division in *TSC2*^−/−^ compared to WT scNETs (Figure 3G), supporting previous reports that TSC2 loss alters cell differentiation^46^ and that during early development, this manifests as accelerated neurogenesis.^47^ Indeed, even at this very early stage of neurodevelopment, scNETs showed a slight increase in the number of MAP2^+^ cells (Figure 3H). Altogether, we demonstrate that our bioprinting approach permits the generation of reproducible scNETs and the manifestation of MCD disease-associated phenotypes in just a few days.

### Rapamycin rescues *TSC2*^−/−^ phenotypes in scNETs

**Figure 4:**
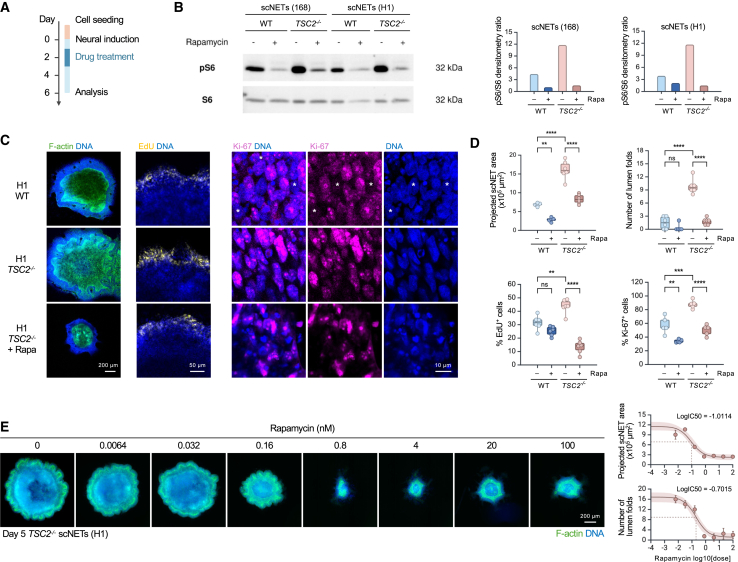
Rapamycin rescues hyperproliferative and folding *TSC2*^−/−^ phenotypes (A) Timeline of drug treatment. (B) Western blots showing S6/pS6 levels of scNETs ± rapamycin. Full blots are shown in Figure S4D. Boxplots show pS6/S6 ratios. (C) Confocal images of day 6 DMSO-treated WT, DMSO-treated *TSC2*^−/−^, and rapamycin-treated *TSC2*^−/−^ scNETs (H1) stained for F-actin (green), DNA (blue), EdU (yellow), and Ki-67 (magenta). Asterisks mark the cells with no detectable Ki-67 expression. (D) Boxplots showing the projected whole area, the number of lumen folds, the percentage of EdU^+^ cells, and the percentage of Ki-67^+^ cells of WT and *TSC2*^−/−^ scNETs (H1) ± rapamycin. Each dot represents an individual micropatterned scNET (results of one representative experiment are shown). Reproducible results were seen in all three biological replicates (*n* ≥ 5 for each biological replicate per genotype and treatment). ∗∗∗∗*p* < 0.00005, ∗∗∗*p* < 0.0005, and ∗∗*p* < 0.005, unpaired t test. (E) Fluorescent images showing *TSC2*^−/−^ scNETs (H1), treated with rapamycin at indicated concentrations since neural induction, stained with F-actin (green) and DNA (blue) (left). IC50 curves showing the projected whole pattern area and the number of lumen folds (mean with standard error of mean) versus the rapamycin dose (right). Scale bars: 200 μm, 50 μm, and 10 μm. See also Figure S4.

Finally, we assessed the sensitivity of the scNET model to reflect phenotypic changes in response to drug treatment. Using levels of phosphorylated ribosomal protein S6 at Ser235/236 (pS6) as an indicator of mTORC1 activity,^26^^,^^48^ we confirmed mTORC1 hyperactivity in *TSC2*^−/−^ scNETs compared to the WT (Figure 4B). Treatment of scNETs with rapamycin (20 nM), an mTORC1 inhibitor that represents the leading clinical treatment of patients with TSC,^49^^,^^50^^,^^51^ for 48 h starting on day 1 (Figure 4A) reduced pS6 levels (Figures 4B and S4D). Additionally, *TSC2*^−/−^ scNETs treated with rapamycin (20 nM) were significantly smaller in size (Figures 4C, 4D, S4A, and S4B) than untreated *TSC2*^−/−^ scNETs and had significantly fewer folds. Ki67 immunostaining and EdU incorporation further showed that rapamycin rescued the increased proliferation observed in untreated *TSC2*^−/−^ scNETs, as both were significantly decreased in treated tissues (Figures 4C and 4D).

To demonstrate the high-content and high-throughput compatibility of our model, we printed ECM droplets in a 96-well plate format and asked if we could determine the rapamycin concentration required to achieve 50% rescue of *TSC2*^−/−^ phenotypes, i.e., the IC50 (Figure 4E). *TSC2*^−/−^ scNETs were treated with increasing rapamycin concentrations using a 5-fold serial dilution. On day 3, graded responses of *TSC2*^−/−^ scNETs to rapamycin in terms of their size and degree of folding were already evident (Figure S4C). Quantitative analyses of the day 5 scNETs revealed that rapamycin inhibited tissue size (IC50 = 0.0974 nM) and the number of folds (IC50 = 0.198 nM) in a dose-dependent manner (Figure 4E). These IC50 values, being in the nanomolar range, indicated that *TSC2*^−/−^ phenotypes were highly sensitive to rapamycin.

In summary, we have reported a versatile bioprinting approach to generate reproducibly sized micropatterned scNETs that show high fidelity to early human brain development. We highlighted the ability of our method to reveal quantifiable disease-associated phenotypes and its compatibility for high-content, high-throughput genetic and chemical compound screens.

## Discussion

In this study, we show that bioprinting can reliably produce ECM droplets of defined diameter to facilitate the generation of micropatterned scNETs. While this work was done using a commercially available extrusion bioprinter, the rise of open-source, low-cost 3D bioprinting technologies and automated pipettes^52^^,^^53^^,^^54^ means that labs will be able to incorporate these models into their workflows more readily. Our bioprinting approach is compatible with any extrusion-based 3D bioprinter with extrusion volume resolution in the nano-to microliter range and with spatial resolution in the range of 0.1–1 millimeter. With a 27G blunt needle, which provides optimal control over droplet size, we can reliably print ∼100 droplets in 3 min, and the droplet diameter can be controlled by adjusting the print pressure and extrusion speed (Figure S1). We found that seeding hPSCs onto droplets of 700–800 μm in diameter reproducibly generated single-lumen scNETs, with full lumen closure occurring in ∼80% of the tissues. This is in line with previous studies showing that 20% of 500-μm-diameter colonies exhibit an open central cavity.^7^

The standardized and reproducible generation of scNETs with physiologically relevant cell organization makes them a unique and accessible tool for studying early neurodevelopment. While many hPSC-derived 2D and 3D *in vitro* neural models exist,^55^ our 6 day protocol can generate NETs containing self-assembled neural progenitor ventricular zones and even β-TubIII^+^ and MAP2^+^ cells with a reproducible structure and in a much shorter time frame and with greater structural fidelity than most existing protocols. This lends the scNET model well to high-throughput drug screening applications. We have demonstrated that matrix droplets can be printed rapidly and in large numbers in a 96-well plate. The bioprinting approach also offers greater flexibility than current tools used to produce growth-restricted surfaces for micropatterning, as we can easily print in different cell culture vessels and assess the effects of different factors, such as ECM substrates, chemical compounds, and culture conditions. While we used the ECM Cultrex in this study for droplet printing, we also observed comparable performance in terms of cell attachment and scNET formation using Matrigel.

Finally, we provide a unique and powerful tool to quantitatively study the early stages of MCD development and to rapidly assess drug responses. We found that *TSC2*^−/−^ scNETs display hyperproliferation and extensive folding, mimicking MCD phenotypes. Although rapamycin treatment significantly suppressed proliferation of *TSC2*^−/−^ scNETs, their size remained larger than that of WT counterparts in the presence of rapamycin, suggesting that TSC2 loss may impact cell proliferation through additional, rapamycin-insensitive pathways. This is in line with previous findings showing evidence of non-canonical TSC pathways (reviewed in Neuman and Henske^56^ and Henske et al.^57^). These findings open a new avenue for future studies employing targeted genetic and pharmacological manipulations to dissect alternative signaling pathways that lead to *TSC2*^−/−^ phenotypes. Our observations of *TSC2*^−/−^ phenotypes in day 6 scNETs reflect those seen in cerebral organoids at a later developmental stage, wherein deletion of the tumor suppressor *PTEN*, another inducer of mTORC1-driven MCDs, leads to increased proliferation, cortical size, and surface folding.^58^^,^^59^ TSC2 deficiency itself has been shown to alter the structural organization of randomly produced neuroectodermal rosettes in culture and brain organoids^47^^,^^60^ and to cause defects of neural tube closure and morphogenesis in mouse models.^61^ Excess cortical folding is also linked to more severe clinical phenotypes in patients with TSC.^14^ Our findings contextualize these earlier observations by suggesting that the mechanisms underlying morphogenic tissue alterations in MCDs begin at very early developmental stages, supporting hypotheses that neural stem cell projections underlie early stages of cortical folding.^62^ A previous organoid-on-a-chip approach to induce geometrically confined early brain development demonstrated a similar folding phenotype, though this was not prominent until the second week following neuroepithelial induction.^9^ This suggests that scNETs may have the potential to capture cortical folding in WT tissues if given more time to develop and that TSC2 deficiency accelerates the normal timeline of tissue folding. To better understand the mechanisms behind cortical folding and other aspects of MCD formation, future work will include the dissociation of scNETs from the culture surface to produce free-floating single-lumen organoids in extended culture.

### Limitations of the study

This study describes a protocol that generates NETs from hPSCs over the span of 6 days, reflecting cellular mechanisms that occur early in brain development during neural tube formation. Currently, scNETs are not cultured past day 6 since the tissues begin to darken and die as the cells become overconfluent. Thus, this current system cannot capture events that occur in later stages of neurodevelopment (e.g., gliogenesis, neuron maturation, or expanded neurogenesis). Additionally, this study used hPSCs with a complete loss of *TSC2*. Future work will involve generating scNETs with hPSCs that carry heterozygous *TSC2* mutations representing a variety of patient-derived variants, as we expect this model will permit insightful genotype-phenotype correlation analyses.

## Resource availability

### Lead contact

Further information and requests for resources and reagents should be directed to the lead contact, Lisa M. Julian (ljulian@sfu.ca).

### Materials availability

This study did not generate new unique reagents.

### Data and code availability

• All datasets used in this study have been summarized in the key resources table.
• The G-code scripts used to generate micropatterned scNETs and a README file are available in the Open Science Framework at https://osf.io/538qa/. The DOI (10.17605/OSF.IO/538QA) is also listed in the key resources table.
• Any additional information required to reanalyze the data reported in this study is available from the lead contact upon request.

## Acknowledgments

We thank Dr. William Stanford for generously providing the hPSC lines. We also thank all members of the Julian lab for helpful discussions and technical support, Dr. Lorena Braid’s lab for use of their Incucyte live-cell microscope, and Dr. Mahmoud Pouladi for helpful scientific discussions. This work was supported by funding from the 10.13039/100009326Cancer Research Society (JULIAN, L-CRS 25551), the 10.13039/501100000038Natural Sciences and Engineering Research Council of Canada (NSERC; JULIAN, L-RGPIN-03965), and the New Frontiers in Research Fund (NFRFE-2023-00824). L.M.J. is a Tier II Canada Research Chair and a Michael Smith Foundation for Health Research/Parkinson Society BC Scholar. L.L. is funded by 10.13039/501100004489Mitacs and the NSERC Alliance program and S.N. by SFU and Phyllis Carter Burr Graduate Fellowships. Graphs and illustrations were created using BioRender.com, including the graphical abstract—created in BioRender. Julian, L. (2025) https://BioRender.com/utwhvlp.

## Author contributions

Conceptualization, N.I.F., K.K.L.W., G.A., and L.M.J.; validation, K.K.L.W. and G.A.; methodology and resources, N.I.F., K.K.L.W., G.A., A.M., L.L., S.N., and L.M.J.; investigation, K.K.L.W., N.I.F., and A.M.; writing – original draft and writing – review & editing, K.K.L.W., N.I.F., G.A., and L.M.J.; supervision, L.M.J.; funding acquisition, L.M.J.

## Declaration of interests

The authors declare no competing interests.

## STAR★Methods

### Key resources table

**Table:** 

REAGENT or RESOURCE	SOURCE	IDENTIFIER
**Antibodies**		

Rabbit anti-PAX6	Proteintech Group	Cat#12323-1-AP; RRID: AB_2159695
Rabbit anti-Ki-67	Proteintech Group	Cat#27309-1-AP: RRID: AB_2756525
Mouse anti-ZO-1	Invitrogen	Cat#33–9100; RRID: AB_2533147
Mouse anti-β-Tubulin III	STEMCELL Technologies	Cat# 60100
Mouse anti-N-cad	Cell Signaling	Cat#14215; RRID: AB_2798427
Chicken anti-MAP2	Millipore Sigma	Cat#AB5543; RRID: AB_571049
Mouse anti-S6	Cell Signaling Technology	Cat#2317; RRID: AB_2238583
Rabbit anti-Phospho-S6	Cell Signaling Technology	Cat#2211S; RRID: AB_331679
IRDye® 800CW Donkey anti-Mouse IgG Secondary Antibody	LICORbio	Cat#926–32212; RRID: AB_621847
IRDye® 680RD Goat anti-Mouse IgG Secondary Antibody	LICORbio	Cat#926–68070; RRID: AB_10956588

**Chemicals, peptides, and recombinant proteins**		

Rapamycin	Cedarlane	Cat#13346-10
Accutase	STEMCELL Technologies	Cat#07922
Hoescht 33342	Enzo	Cat#ENZ-52401
DMSO	Sigma	Cat#D2438
hESC matrigel	Corning	Cat#354277
DMEM-F12	Gibco	Cat#11320-033
Neurobasal	Gibco	Cat#21103-049
N2	Gibco	Cat#17502-048
B27	Gibco	Cat#17504-044
MEM Non-Essential Amino Acids Solution (100X)	Gibco	Cat#11140-050
SB 431542	STEMCELL Technologies	Cat#72234
LDN 193189 (2HCl)	STEMCELL Technologies	Cat#72147
mTeSR plus	STEMCELL Technologies	Cat#100-0276
HBSS	Gibco	Cat#14175-095
ReLeSR	STEMCELL Technologies	Cat#100-0483
ROCK inhibitor	STEMCELL Technologies	Cat#72304
Prolong antifade	ThermoFisher	Cat#P36984
Trypan blue	STEMCELL Technologies	Cat#07050
Cultrex	BioTechne	Cat#BME001-10
Glutamax	Gibco	Cat#35050-061
Pierce Protease Inhibitor Tablets	ThermoFisher	Cat#A32963
Phosphatase Inhibitor Cocktail V	Millipore Sigma	Cat#524629
RIPA Buffer	Sigma-Aldrich	Cat#R0278
OneTaq Quick-Load 2X Master Mix	New England Biolabs	Cat#M0486
PmeI	New England Biolabs	Cat#R0560
Cutsmart Buffer	New England Biolabs	Cat#B6004S
10× BlueJuice Gel Loading Buffer	Thermo Fisher	Cat#10816015

**Critical commercial assays**		

PowerUp SYBR Green Master Mix	Applied Biosystems	Cat#A25741
Quick-DNA/RNA Microprep Plus Kit	Zymo Research	Cat#D7005
MycoFluor Mycoplasma Detection Kit	Invitrogen	Cat#M7006
High-Capacity cDNA Reverse Transcription Kit	Applied Biosystems	Cat#4368814
Pierce™ BCA Protein Assay Kit	ThermoFisher	Cat#23227

**Experimental models: Cell lines**		

168 WT	Delaney et al.^26^	N/A
168 TSC2 KO	Delaney et al.^26^	N/A
H1 WT	Delaney et al.^26^	N/A
H1 TSC2 KO	Delaney et al.^26^	N/A

**Oligonucleotides**		

qPCR primers for *TBP*	Integrated DNA Technologies	See Table S1 for primer sequence.
PCR primers for *TSC2*	Delaney et al.^26^	See Table S1 for primer sequence.
qPCR primers for *PAX6*	Integrated DNA Technologies	See Table S1 for primer sequence.
qPCR primers for *FOXG1*	Integrated DNA Technologies	See Table S1 for primer sequence.
qPCR primers for *NKX2-1*	Integrated DNA Technologies	See Table S1 for primer sequence.
qPCR primers for *EN1*	Integrated DNA Technologies	See Table S1 for primer sequence.
qPCR primers for *OCT4*	Integrated DNA Technologies	See Table S1 for primer sequence.

**Software and algorithms**		

Zen	Carl Zeiss	RRID: SCR_013672
ImageJ	National Institutes of Health	RRID: SCR_003070
Graphpad prism	Graphpad	RRID: SCR_002798
BioRender	Biorender	RRID:SCR_018361
Image Lab Software	Bio-Rad	RRID: SCR_014210

**Deposited data**		

Custom g-code	This study	Open Science Framework: https://doi.org/10.17605/OSF.IO/538QA

**Other**		

Incucyte	Sartorius	RRID: SCR_023147
Glass coverslips	VWR	Cat#:48393048
Thermanox coverslips	NUNC	Cat#:174969
CELLINK BIOX6 Bioprinter	CELLINK	N/A
ChemiDoc MP Imaging System	Bio-Rad	RRID:SCR_019037

### Experimental model and study participant details

#### Cell models

Human PSC lines used in this study were 168 id2 (iPSC, male) and H1 (hESC, male; WiCell WA01). The control 168 line was generated previously and reported in.^54^ Isogenic *TSC2*^−/−^ 168 and H1 lines were generated previously, and their *TSC2* genotype status authenticated previously.^27^ hPSCs were maintained in a standard feeder-free culture system using mTeSR Plus (mTeSR^+^) medium (STEMCELL Technologies 100–0276) on diluted Matrigel hESC-Qualified Matrix (Corning 354277) prepared according to lot-specific manufacturer instructions, cultured in 6-well tissue culture plates at 37°C, 5% CO_2_. Cells were passaged by incubation with ReLeSR (STEMCELL Technologies) for 30 s followed by aspiration and incubation of the plate in a 37°C incubator (5% CO_2_) for 4 min, then gentle resuspension in mTESR+ and transfer to a new pre-coated plate by wide-bore pipette. hPSC cultures were visually examined at each passage to ensure the absence of differentiated mesenchymal-like cells and abnormal cells were removed by scraping with a pipette tip. All cell lines tested negative for mycoplasma using the MycoFluor Mycoplasma Detection Kit (ThermoFisher M7006).

### Method details

#### 3D printing of extracellular matrix

Extracellular matrix (ECM) droplets were printed using a CELLINK BIO ×6 bioprinter onto Nunc Thermanox coverslips (Thermo Scientific #174969) in a 24-well tissue culture plate. Empty wells in the plates were filled with sterile water, creating a humid environment to prevent droplet evaporation. Cultrex (R&D Systems #BME001-10) prepared at 1.5× concentration was added into a BIO ×6 print cartridge. A 27-gauge needle was then fitted to the print cartridge along with the air adapter connector. The assembled cartridge was then inserted into the print position of the printer. The BIO ×6 printer software used was DNA studio 3 with the print protocol setup using the Model function directed by G-code script. Print pressure was set to 15 kPa, unless otherwise indicated. To minimize droplet evaporation, print bed temperature was set to 14°C and the internal fan was disabled. After print head calibration, the print job was released. Once ECM printing was complete, the plate was sealed with parafilm and refrigerated for at least 2 h before cell seeding. The vessels with printed droplets were stored at 4°C for up to two weeks before use. Droplet size was controlled with the g-code script at a diameter of 800 μm. Before cell seeding, the wells with printed ECM droplets were spray washed with PBS. The G-code scripts used to generate micropatterns and a README document have been deposited in the Open Science Framework https://osf.io/538qa/.

#### Generation of scNETs

Colonies of human PS cells grown to a confluence o f >70% were dissociated using Accutase (STEMCELL Technologies #07920) at 37°C for 6 min. Single cells were resuspended in mTeSR^+^ supplemented with 10 μM ROCK inhibitor Y-27632 (STEMCELL Technologies), followed by seeding at the indicated density onto the Cultrex-coated coverslips. After 2 h, the ROCK inhibitor was removed by replacing the media with fresh mTeSR^+^. Once the hPSCs fully covered the printed droplet area, typically within 24–48 h, neural induction and lumenogenesis (Day 0) was initiated. Cells were treated with neural induction media (NIM) containing DMEM/F12 (Gibco, 11320-033), Neurobasal (Gibco 11320-033), 1% N2 (Gibco, 17502-048), 2% B27 (Gibco, 17504-044), 1× Glutamax (Gibco 35050-061), 1% MEM (Gibco, 11140-050) with dual SMAD inhibitors SB-431542 (STEMCELL Technologies, 72234, 10 μM) and LDN (STEMCELL Technologies, 72147, 500nM) with 4% Matrigel. A full media change was done every two days post-neural induction using NIM with 2% Matrigel. For rapamycin treatment, the drug was added to the cells on Day 1 for 48 h at 20 nM, unless otherwise indicated.

#### Immunostaining of scNETs

Cells were rinsed in PBS and fixed in 4% PFA in PBS for 15 min at room temperature. The cells were then washed with PBS with 0.1% Triton X-100 (PBST), followed by incubation with 5% normal goat serum (NGS) in PBST for 1 h at room temperature. After blocking, the cells were incubated with primary antibodies overnight at 4°C. Primary antibodies used include rabbit anti-PAX6 (1:1000; Proteintech #12323-1-AP), rabbit anti-Ki-67 (1:200; Proteintech #27309-1-AP), mouse anti-ZO-1 (1:200; Invitrogen #33–9100), mouse anti-β-Tubulin III (1:500; STEMCELL Technologies #60100), mouse anti-N-cad (1:500; Cell Signaling #14215), chicken anti-MAP2 (1:5000; Millipore Sigma #AB5543). After washing with PBST, the cells were incubated with secondary antibodies, phalloidin and DAPI in the dark overnight at 4°C, followed by a final wash, mounting and imaging.

#### Image acquisition and processing

Images were taken on a laser scanning microscope (Zeiss LSM880 with Airyscan) and processed by ImageJ. Time-lapse imaging was performed on the Incucyte Live-Cell Analysis System (Sartorius).

#### EdU assay

EdU assay was performed using Click-iT EdU Cell Proliferation Kit for Imaging, Alexa Fluor 488 dye (Invitrogen #C10337). Briefly, scNETs were treated with 10 μM of EdU for 2 h, followed by fixation and PBST wash. Click chemistry was performed by incubating the fixed samples with the Click-iT reaction mix cocktail for 30 min at room temperature in the dark. After PBST wash, the samples were blocked with NGS for downstream immunostaining.

#### RT-qPCR

Total RNA isolation was performed using Quick-DNA/RNA Microprep Plus Kit (Zymo Research D7005), followed by cDNA synthesis using High-Capacity cDNA Reverse Transcription Kit (Applied Biosystems 4368814). RT-qPCR was performed using PowerUp SYBR Green Master Mix (Applied Biosystems A25742) on CFX384 Real-Time System (Bio-Rad). See Table S1 for primers used.

#### PCR

*TSC2* expression was knocked out by introduction of a frameshift 35-base stop codon sequence at *TSC2* exon 3, which is confirmed by PmeI digestion. Genomic DNA isolation was performed using the Quick-DNA/RNA Microprep Plus Kit (Zymo Research D7005). PCR reaction was performed using OneTaq Quick-load 2× Master Mix (New England Biolabs M0486) with 300 ng of isolated template DNA and TSC2 primers with cycling parameters of: 94°C for 30 s, 25× [94°C 20s, 56.5°C 30 s, 68°C 30s], 68°C for 5 min, 4°C for ∞. The PCR product then underwent a restriction digest reaction with PmeI (New England Biolabs R0560) in CutSmart Buffer (New England Biolabs B6004S) for 1 h at 37°C, followed by heat inactivation at 65°C for 20 min. Samples were loaded in 10× BlueJuice Gel Loading Buffer (Thermo Fisher 10816015) and run for 30 min at 100V on a 1% agarose gel and imaged using ethidium bromide on the ChemiDoc MP Imaging System (Bio-Rad). See Table S1 for primers used.

#### Western blotting

Cells were lysed with 1× RIPA Buffer (Sigma-Aldrich R0278), supplemented with Pierce Protease Inhibitor (ThermoFisher A32963) and Phosphatase Inhibitor Cocktail (Millipore Sigma 524629). Protein lysates with 1X SDS sample buffer were resolved on SDS/PAGE and then transferred to nitrocellulose membranes. Membranes were blocked with 5% bovine serum albumin for 1 h before overnight primary antibody incubation. Subsequently, the blots were incubated in fluorescent secondary antibodies for 2 h. Blots were then imaged on the ChemiDoc MP Imaging System (Bio-Rad) and target protein was quantified using Image Lab Software (Bio-Rad). See key resources table for antibodies used.

### Quantification and statistical analysis

Quantifications of projected whole pattern area, lumen area, lumen circularity, the number of lumen folds, the percentages of EdU^+^ and Ki-67^+^ cells were performed using ImageJ. We used the built-in *Line Selection* tools (segmented or freehand), to manually outline the whole scNET area and the lumen. Using the *Measure* tool, we determined the areas of the whole pattern and lumen, as well as the lumen circularity. We manually counted the number of lumen folds characterized by formation of gyrus- and sulcus-like structures. To calculate the percentages of EdU^+^ and Ki-67^+^ cells, we first randomly selected multiple neuroepithelial areas of the scNETs and determined the background signals of EdU and Ki-67. Cells were deemed EdU^+^ and Ki-67^+^ if their signals were higher than the background. Total cells were counted using DNA staining. The percentages were determined by the ratio of the number of EdU^+^/Ki-67^+^ cells to the number of total cells. To determine the success rate of scNET formation, we calculated the ratio of the number of scNETs with a lumen structure formed by day 6 to the number of ECM droplets printed, based on three to four independent biological replicates, each containing at least two replicate wells. Each well contained at least six ECM droplets. This assay revealed the variability between wells as well as across replicates. Data were presented by mean ± SEM obtained from at least two biological replicates. *p* values were determined by unpaired t tests. *p* < 0.05 was considered statistically significant.

## Supplemental information

Video S3. Time-lapse imaging of *TSC2*^−/−^ scNET development, related to Figure 3

Document S2. Article plus supplemental information
