# Supplementary material for: High-throughput bioprinting to produce micropatterned neuroepithelial tissues and model TSC2-deficient brain malformations
Source: Cell Rep Methods. 2025 Sep 17;5(10):101177. doi: 10.1016/j.crmeth.2025.101177 (PMC12570318; doi:10.1016/j.crmeth.2025.101177)
Supplement: Document S1. Figures S1–S4 and Table S1 [file mmc1.pdf]

Cell Reports Methods, Volume *5*

Supplemental information

High-throughput bioprinting to produce micropatterned neuroepithelial tissues and model TSC2-deficient brain malformations

Negin Imani Farahani, Kenneth Kin Lam Wong, George Allen, Abhimanyu Minhas, Lisa Lin, Shama Nazir, and Lisa M. Julian

Supplementary Figures

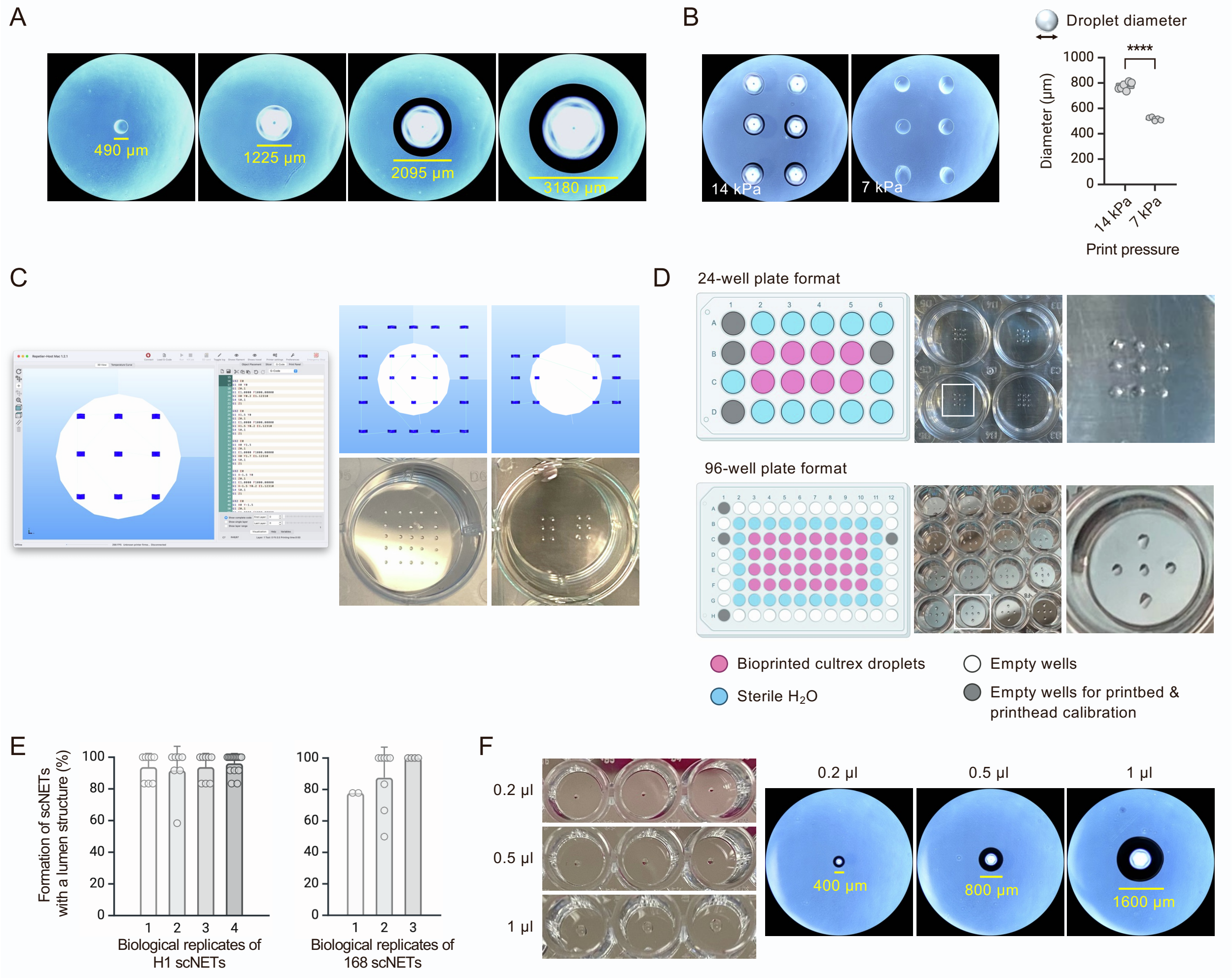

Figure S1. Programmable bioprinting of geometrically-defined ECM droplets at assigned positions. Related to Figure 1.

(A) Brightfield images showing bioprinted ECM droplets of indicated diameters.

(B) Brightfield images showing bioprinted ECM droplets using printhead pressure at either 14 or 7 kPa (left). Boxplot showing the droplet diameter at the indicated pressure. Note that we were able to consistently print arrays of ECM droplets of a similar size. $****p < 0.00005$, unpaired *t*-test.

(C) Screenshots of Repetier-Host showing programmable printing arrangements using G-code (left). The blue rectangles in the G-code simulation viewer represent individual ECM droplets to be printed. Two additional screenshots were shown to illustrate that we can use G-code to control the number and the position of ECM droplets in a customizable and precise manner (right).

(D) Bioprinting in 24-well (left) and 96-well (right) plate formats, with photos and zoom-in views showing the droplets. Wells in grey were used for print bed and printhead calibrations. Wells in blue were filled with sterile water to minimize droplet evaporation. Wells in red were bioprinted with ECM droplets. Wells in both grey and white were empty.

(E) Bar graphs showing the success rate of the formation of scNETs with a lumen structure (%) in independent biological replicates (four replicates for H1 hESC lines and three for 168 iPSC lines), calculated by the ratio of the number of scNETs formed by day 6 to the number of ECM droplets printed. Each dot represents the success rate of one single well, each well containing at least six ECM droplets. At least two replicate wells were included in each biological replicate. Error bars indicate standard deviations.

(F) Manual droplet deposition, which is low-throughput and lacks spatial precision, in a 96-well plate using a P2 pipette, with photos showing single droplets of indicated volumes (left) and brightfield images showing the droplet diameter (right).

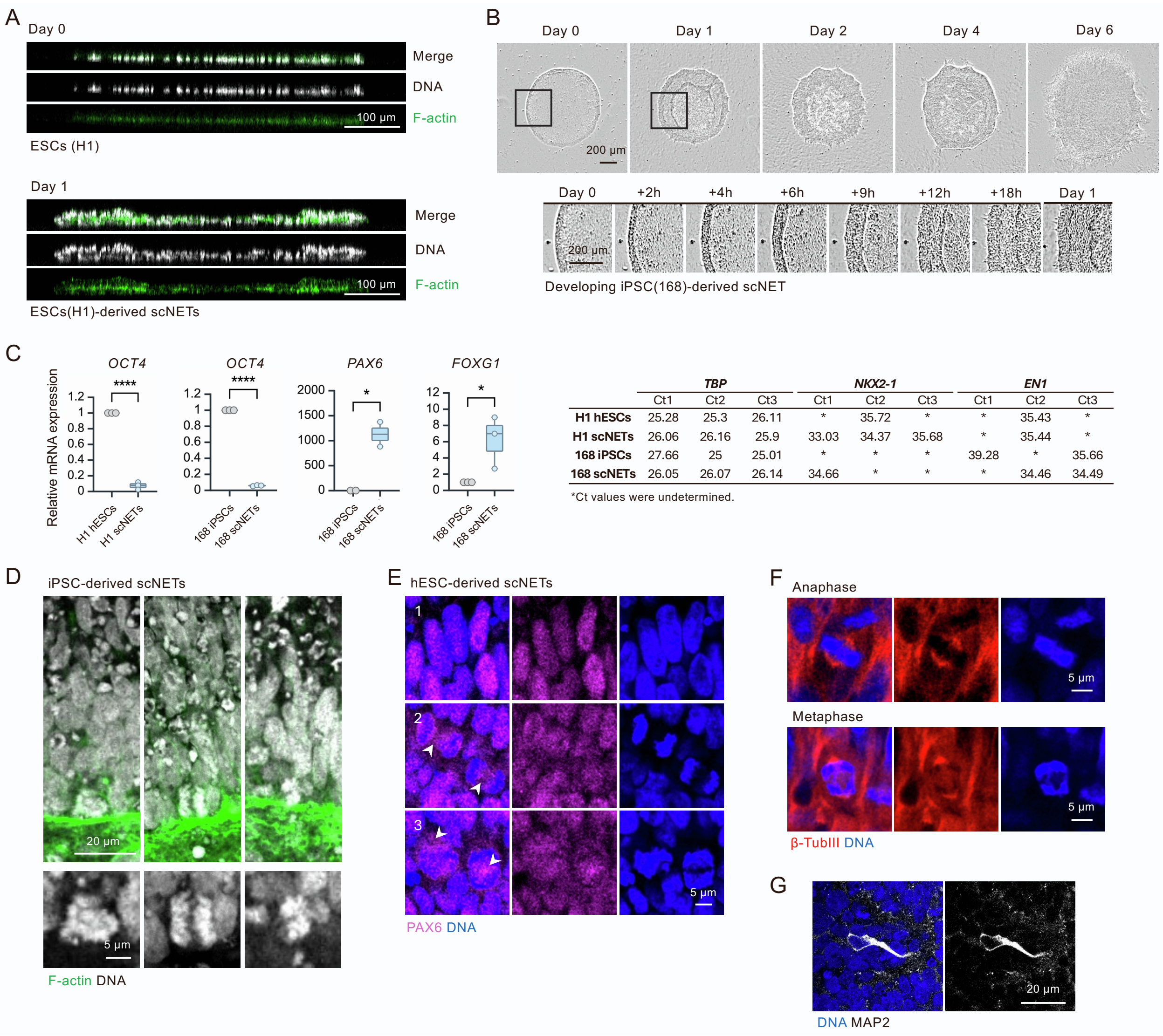

	TBP			*NKX2-1*			*EN1*		
	Ct1	Ct2	Ct3	Ct1	Ct2	Ct3	Ct1	Ct2	Ct3
**H1 hESCs**	25.28	25.3	26.11	*	35.72	*	*	35.43	*
**H1 scNETs**	26.06	26.16	25.9	33.03	34.37	35.68	*	35.44	*
**168 iPSCs**	27.66	25	25.01	*	*	*	39.28	*	35.66
**168 scNETs**	26.05	26.07	26.14	34.66	*	*	*	34.46	34.49

Figure S2. Developing scNETs self-assemble into 3D neuroepithelial tissue-like structure with neurogenic potential. Related to Figure 1 and Figure 2.

(A) Confocal images of undifferentiated hESCs (H1) on day 0 and developing scNETs (H1) on day 1, stained for F-actin (green) and DNA (grey).

(B) Phase-contrast time-lapse imaging of the development of scNET derived from iPSCs (168) starting from day 0 till day 6 (left). Zoom-in views of the insets showing the edge thickening that took place between day 0 and day 1. Scale bars indicate 200 µm. Refer to **Video 1**.

(C) Left: Boxplots showing relative mRNA expression levels of *OCT4*, *PAX6* and *FOXG1* of day 6 scNETs and the undifferentiated PSCs (H1: hESCs; 168: iPSCs). ****p < 0.00005; *p < 0.05, unpaired *t*-test. Right: RT-qPCR data showing the values of threshold cycle (Ct) of the indicated genes of the day 6 scNETs and the undifferentiated PSCs (H1: hESCs; 168: iPSCs). Ct1, Ct2 and Ct3 represent three technical replicates in one biological replicate. Comparable results were obtained in three independent biological replicates. Asterisks indicate Ct values that could not be determined.

(D) Confocal images of the neuroepithelium-like layer of scNETs derived from iPSCs (168), stained for F-actin (green) and DNA (blue). Scale bars 20 µm and 5 µm are shown.

(E) Zoom-in confocal images of **Figure 2C** showing the non-dividing **(row 1)** and dividing cells **(rows 2-3)** in day 6 scNETs (H1), stained for PAX6 (magenta) and DNA (blue) (left). Arrowheads mark the region where PAX6 showed little overlap with DNA in mitotic cells. Scale bar indicates 5 µm.

(F) Confocal images of dividing NPCs in anaphase and metaphase in day 6 scNETs, stained for β-TubIII (red) and DNA (blue). Note that β-TubIII appears to mark the spindle poles in dividing cells. Scale bar indicates 5 μm.
(G) Confocal images of a $MAP2^+$ cell in scNETs derived from iPSCs (168), stained for MAP2 (grey) and DNA (blue). Scale bar indicates 20 μm.

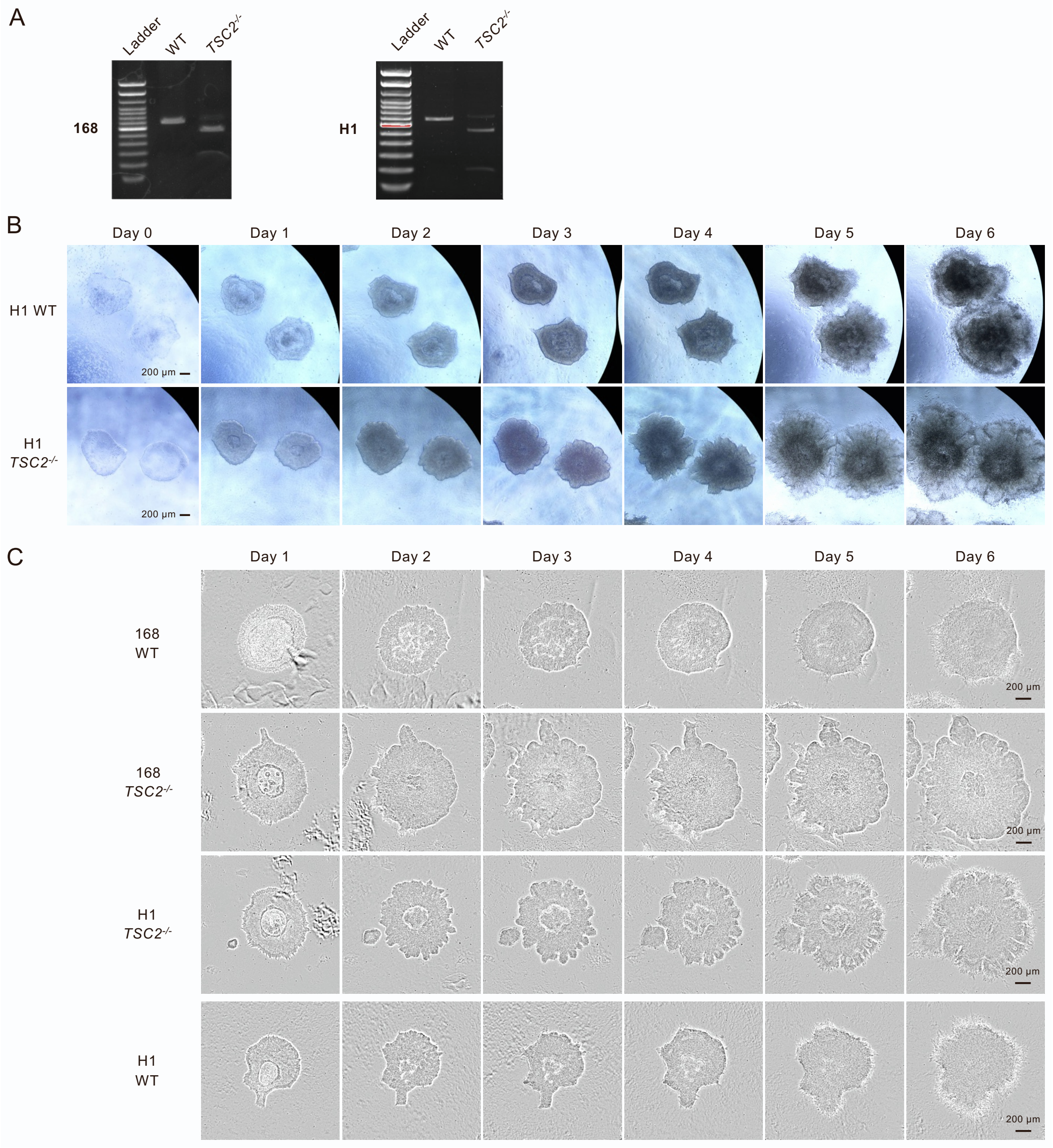

Figure S3. ***TSC2**$^{-/-}$* **scNETs display cortical folding-like phenotype as early as on day 2. Related to Figure 3.**
(A) Verification of *TSC2*$^{-/-}$ cell lines (hESC H1 and iPSC 168) by PCR. See method section for details.
(B) Brightfield images of developing scNETs derived from WT hESCs (H1) (top row) and those derived from *TSC2*$^{-/-}$ hESCs (H1).
(C) Phase-contrast time-lapse imaging of developing scNETs derived from WT iPSCs (168) (1st row), *TSC2*$^{-/-}$ iPSCs (168) (2nd row), WT hESCs (H1) (3rd row) and *TSC2*$^{-/-}$ hESCs (H1) (4th row). Note that even though the initial shape of the WT scNET (H1) was irregular, it did not cause folding as extensive as the knockout counterpart.

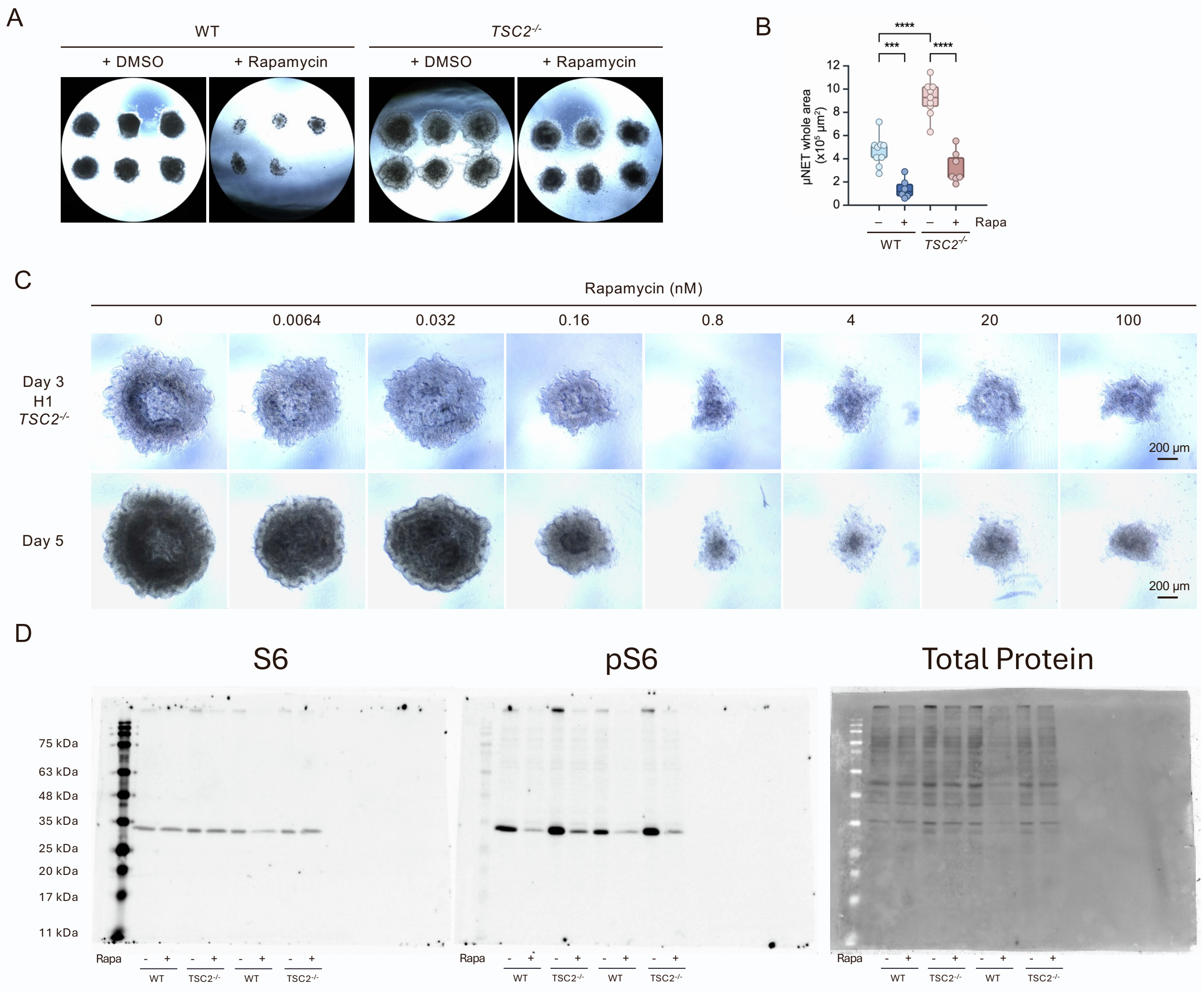

Figure S4. ***TSC2***$^{-/-}$ **scNET phenotypes can be rescued by rapamycin. Related to Figure 4.**
(A) Brightfield images showing scNETs derived from WT hESCs (H1) and those *TSC2*$^{-/-}$ hESCs (H1), treated with either DMSO or rapamycin.
(B) Boxplots showing the projected whole area WT scNETs (168) and *TSC2*$^{-/-}$ scNETs (168), treated with and without rapamycin. ****$p < 0.00005$, ***$p < 0.0005$, unpaired *t*-test.
(C) Brightfield images of day 3 and day 5 *TSC2*$^{-/-}$ scNETs (H1) treated without or with rapamycin at the indicated concentration. Scale bar indicates 200 µm.
(D) Full blot data for **Figure 4B**.

Supplementary Table

Table S1. Primer sequences used in qPCR and PCR assays. Related to STAR Methods.

Oligonucleotides		
qPCR primers for *TBP*<br>Forward: 5'-CAAGAACTTAGCTGGAAAACCC-3'<br>Reverse: 5'-GATAAGAGAGCCACGAACCAC-3'	Integrated DNA Technologies	Hs.PT.39a.22214825
PCR primers for *TSC2*<br>Forward: 5'-TCC TCG GGA TGG AGC AGT AA-3'<br>Reverse: 5'-TGC AAA CCA GAT CAT CGG CA-3'	*Delaney et al., 2020*	
qPCR primers for *PAX6*<br>Forward: 5'-GACACCACCGAGCTGATTC-3'<br>Reverse: 5'-ATTTCAGAGCCCCATATTCGAG-3'	Integrated DNA Technologies	Hs.PT.58.3002797
qPCR primers for *FOXG1*<br>Forward: 5'-CGTCCACCATATAGTTCCATGA-3'<br>Reverse: 5'-TGACTGCTTTGCCATTTCATTC-3'	Integrated DNA Technologies	Hs.PT.58.26906112.g
qPCR primers for *NKX2-1*<br>Forward: 5'-TGCCGCTCATGTTCATGC-3'<br>Reverse: 5'-CAGGACACCATGAGGAACAG-3'	Integrated DNA Technologies	Hs.PT.58.2461055
qPCR primers for *EN1*<br>Forward: 5'-CGCAGCAGCCTCTCGTATG-3'<br>Reverse: 5'-CCTGGAACTCCGCCTTGAG-3'	Integrated DNA Technologies	
qPCR primers for *OCT4*<br>Forward: 5'-GTTGGAGGGAAGGTGAAGTTC-3'<br>Reverse: 5'-TGTGTCTATCTACTGTGTCCCA-3'	Integrated DNA Technologies	
